# Upregulation of circ_0008812 and circ_0001583 predicts poor prognosis and promotes breast cancer proliferation

**DOI:** 10.3389/fmolb.2022.1017036

**Published:** 2022-09-19

**Authors:** Hong Lin, Fangyi Long, Xiqian Zhang, Pinghan Wang, Ting Wang

**Affiliations:** ^1^ Department of Clinical Research, Sichuan Cancer Hospital and Institution, Sichuan Cancer Center, School of Medicine, University of Electronic Science and Technology of China, Chengdu, China; ^2^ Laboratory Medicine Center, Sichuan Provincial Maternity and Child Health Care Hospital, Affiliated Women’s and Children’s Hospital of Chengdu Medical College, Chengdu Medical College, Chengdu, China; ^3^ Department of Pharmacy, The Third People’s Hospital of Chengdu & College of Medicine, Southwest Jiaotong University, Chengdu, China

**Keywords:** breast cancer, circ_0008812, circ_0001583, competing endogenous RNA, prognosis, biomarker

## Abstract

**Background:** Accumulating evidence suggests that circular RNAs (circRNAs) are highly correlated with tumor progression and pathogenesis in breast cancer. Whereas, their regulatory roles and corresponding mechanisms in breast cancer are still not exhaustive. Thus, we intended to establish circRNA-mediated competive endogenous RNA (ceRNA) network to uncover the possible roles and clinical implications of circRNAs in breast cancer.

**Methods:** Microarray and RNA-sequencing (RNA-seq) data were download from GEO and TCGA database to screen for differentially expressed RNAs (DEcircRNAs, DEmiRNAs, DEmRNAs) in breast cancer. By implementing online databases, we established ceRNA networks, performed gene set enrichment analysis, constructed protein-protein interaction (PPI) networks, and assessed the expression levels and prognostic significance of hub genes. Subsequently, we explored the functions of prognosis-related genes and constructed gene-drug interaction networks. Finally, the functional roles of DEcircRNAs in breast cancer were revealed *via* MTT and colony formation assay.

**Results:** Based on the identified 8 DEcircRNAs, 25 miRNAs and 216 mRNAs, a ceRNA regulatory network was established. Further analysis revealed that prominent enrichments were transcription factor binding, transforming growth factor-beta (TGF-β) and Apelin signaling pathway etc. PPI network and survival curves analysis showed that elevated levels of hub genes (RACGAP1 and KPNA2) were associated with poorer prognosis. They were found to be positively relevant to cell cycle and proliferation. Then a prognostic sub-network of ceRNA was constructed, consisting of 2 circRNAs, 4 miRNAs and 2 mRNAs. The gene-drug interaction network showed that numerous drugs could regulate the expression of these two prognosis-related genes. Functional experiments showed that depletion of circ_0008812 and circ_0001583 could significantly inhibit the proliferation of MCF-7 cells.

**Conclusion:** Our study constructed 4 prognostic regulatory axes that are significantly correlated with tumor prognosis in breast cancer patients, and uncover the roles of circ_0008812 and circ_0001583 in breast cancer, providing a new perspective into the molecular mechanisms of breast cancer pathogenesis.

## Introduction

Breast cancer has become the most prevalent malignancy in women worldwide, and the leading cause of cancer-related death in women. According to the latest evidence, new cases of breast cancer in 2020 make up 30% of all malignant tumors worldwide (Siegel et al., 2021). In the past few decades, the treatment of breast cancer has made great progress and mainly includes chemotherapy, radiotherapy, surgery, endocrine therapy, immunotherapy and targeted therapy (Waks and Winer, 2019). Despite these advances, breast cancer still keeps high incidence and mortality worldwide due to high intratumor heterogeneity, which drives tumor metastasis, recurrence, chemo- and radio-resistance (Wang and Fang, 2018; Tomar et al., 2020). Therefore, identifying novel molecular targets is of great significance to reduce breast cancer mortality and improve clinical efficacy.

Circular RNA (circRNA), generated from precursor mRNA (pre-mRNA), has a single-stranded covalently closed circular structure (Jahani et al., 2020). According to differently structural composition, circRNAs can be simply divided into three categories, including exonic circRNAs (ecRNAs), intronic circRNAs (ciRNAs) and exon-intron circRNAs (EIciRNA) (Li et al., 2020a). The mechanisms of forming circRNAs are relatively complicated and has not been fully elucidated. At present, there are three main models to explain their formation mechanisms: lariat-driven, intron pairing-driven and RNA-binding protein mediated circularization (Tang et al., 2021). As a novel type of non-coding RNAs, they are conserved in various species, with high stability, tissue specificity and relatively high expression levels (Kristensen et al., 2019). These characteristics confer circRNAs the potential to be ideal markers for the diagnosis and prognosis of multiple diseases. Different classes of circRNAs appear to have distinct subcellular localizations. The majority of ecRNAs are located in the cytoplasm, nevertheless, ciRNAs and EIcircRNAs are predominantly presented in the nucleus (Zhou et al., 2021). In 2011, the competing endogenous RNA (ceRNA) hypothesis was proposed, arguing that circRNAs could act as microRNA (miRNA) sponges to modulate the downstream target genes (Salmena et al., 2011; Qu et al., 2018). In addition, numerous studies have suggested that circRNAs were involved in the occurrence and development of various cancers. For instance, Zhao et al. (2020) found that circACAP2 could sponge miR-29a/b-3p and modulate COL5A1 to promote breast cancer progression. CircNFATC3 could promote cervical cancer development via utilizing miR-9-5p to up-regulate SDC2 expression (Ma et al., 2021). However, the functions of circRNAs in cancer have not been fully elucidated, and more efficient detection methods of circRNAs and further research on the roles of circRNAs in cancer are needed.

To better uncover the regulatory roles of circRNAs in breast cancer, we identified the expression patterns of circRNAs, miRNAs and mRNAs in breast cancer using data obtained from Gene Expression Omnibus (GEO) and The Cancer Genome Atlas (TCGA) database. We then predicted the circRNAs-miRNAs sponge and miRNA-targeted mRNAs utilizing multiple online databases, and established a ceRNA regulatory network. To better elucidate the function and mechanisms of circRNAs in breast cancer, we subsequently performed comprehensive analysis, including GO and KEGG enrichment, PPI network construction, hub genes identification, survival analysis, gene-drug interaction network construction and so on. Finally, we constructed 4 prognostic regulatory axes. The functional roles of circ_0008812 and circ_0001583, which belong to these prognostic regulatory axes, were further explored by MTT and colony formation assay. These results could help us to reveal the possible roles and clinical implications of circRNAs in breast cancer, and identify new biomarkers and therapeutic targets for breast cancer.

## Materials and methods

### Data acquisition

**FIGURE 1:**
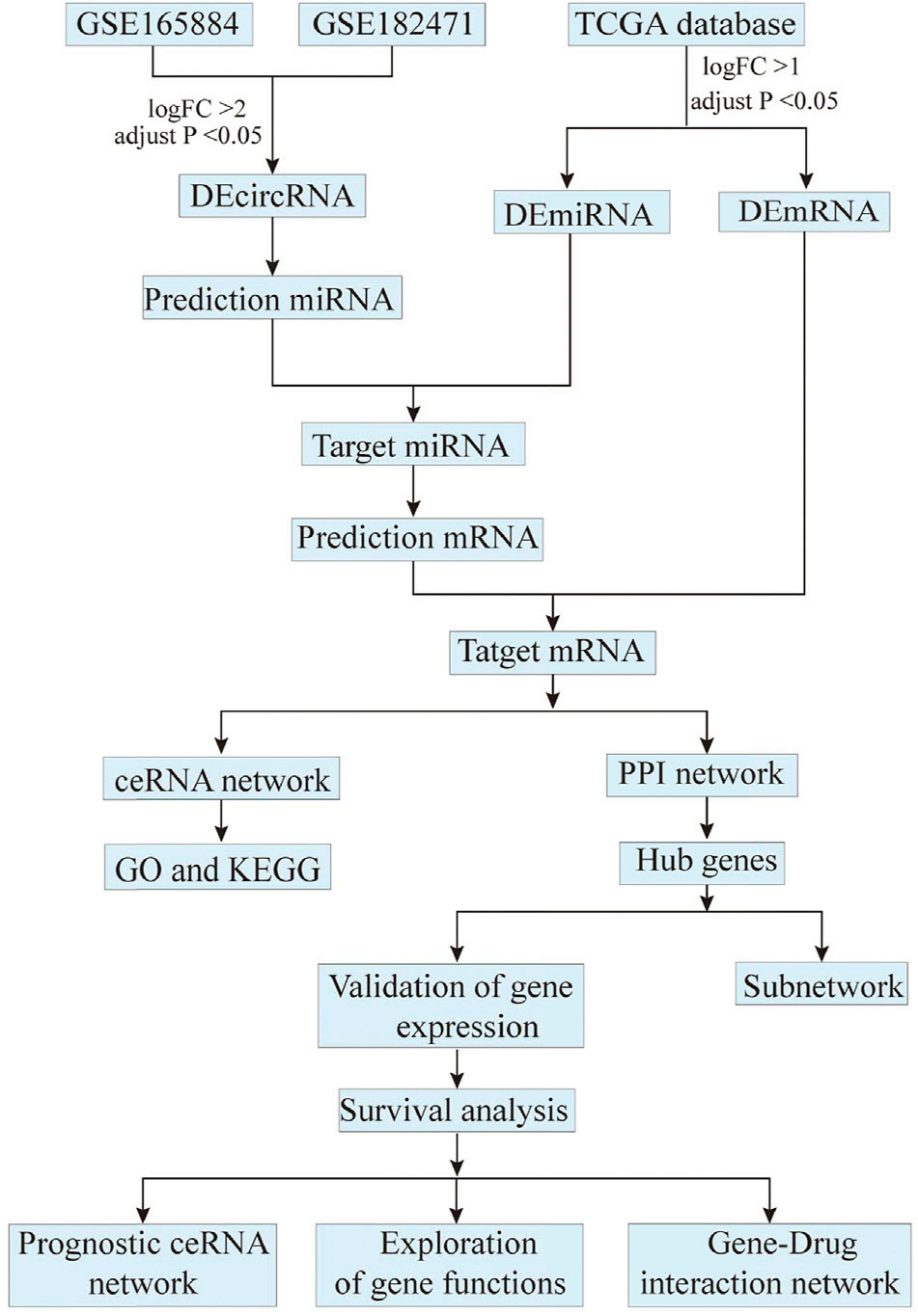
The flowchart of entire analysis process.

Eligible circRNA expression profiles in breast cancer were obtained from GEO database (https://www.ncbi.nlm.nih.gov/geo). The filter criteria were as follows: 1) including breast cancer and adjacent normal tissues, 2) most recent microarray expression profiles. Finally, 2 datasets (GSE165884 and GSE182471) were selected. GSE165884 contains 4 paired cancer and normal tissues. The GSE182471 contains 5 paired cancer and normal tissues. Besides, miRNA (containing 104 normal and 1,103 cancer tissues) and mRNA (containing 113 normal and 1,109 cancer tissues) expression profiles were downloaded from the TCGA database (https://portal.gdc.cancer.gov). The details of these datasets from GEO and TCGA database were exhibited in Table 1. The entire analysis process after data acquisition was shown in Figure 1.

**TABLE 1:** Detailed information of datasets in this study.

Dataset	Platform	Sample		RNA type	Tumor type
		Normal	Tumor		
GSE165884	GPL21825	4	4	circRNA	Breast cancer
GSE182471	GPL21825	5	5	circRNA	Breast cancer
TCGA miRNA		104	1,103	miRNA	Breast cancer
TCGA mRNA		113	1,109	mRNA	Breast cancer

### Differential expression analysis

Differentially expressed circRNAs (DEcircRNAs) were screened by the R “limma” package. Selection criteria were |log2 fold change (FC)|>2 and adjusted *p* < 0.05. Next, the identified results were intersected to construct a heatmap of overlapping circRNAs using the pheatmap package. Additionally, the “edgeR” package was utilized to screen differentially expressed miRNAs (DEmiRNAs) and differentially expressed mRNAs (DEmRNAs) with a filter standard of |log2 FC|>1 and adjusted *p* < 0.05.

### Construction of competive endogenous RNA regulatory network

To further illustrate the mechanism by which circRNAs regulate mRNA, a circRNA-miRNA-mRNA network was constructed. Firstly, CSCD (Xia et al., 2018), (https://gb.whu.edu.cn/CSCD) and CircInteractome (Dudekula et al., 2016), (https://circinteractome.nia.nih.gov) databases were used to predict potential sponge miRNAs of DEcircRNAs. The predicted miRNAs were then identified according to the DEmiRNAs in the TCGA database, and only overlapping miRNAs were considered as candidate miRNAs. Moreover, the target mRNAs were predicted based on miRDB (Chen and Wang, 2020) (http://www.mirdb.org), TargetScan (Lewis et al., 2005) (http://www.targetscan.org/vert_72) as well as miRTarBase (Huang et al., 2020) (http://miRTarBase.cuhk.edu.cn) database. Only the mRNAs recognized by all three databases were further intersected with DEmRNAs to obtain candidate mRNAs. Finally, based on the predicted relationship between circRNA-miRNA and miRNA-mRNA, we established a ceRNA regulatory network and visualized it with the help of Cytoscape 3.8.2 software.

### Functional enrichment analysis

To investigate the regulatory roles of mRNAs in the ceRNA network, we performed a comprehensive analysis including Gene Ontology (GO) and Kyoto Encyclopedia of Genes and Genomes (KEGG) pathway analysis using Metascape online database (Zhou et al., 2019) (https://metascape.org), and visualized through bioinformatics online tool (http://www.bioinformatics.com.cn). The cut-off criterion was *p* < 0.05.

### Construction of protein-protein interaction network

STRING (Szklarczyk et al., 2017) (https://string-db.org) database was used to unveil potential interactions among candidate mRNAs by constructing PPI networks. A composite score > 0.7 was selected as the extraction cut-off criterion for PPI pairs. And the PPI network was visualized with Cytoscape. Next, in order to search for the hub genes in the oncogenic process of breast cancer, the top 10 hub genes of PPI network were screened by the CytoHubba plugin.

### Validation of hub gene expression and survival analysis

To explore hub gene expression in breast cancer, we validated the mRNA levels of hub genes in UALCAN database (Chandrashekar et al., 2017) (http://ualcan.path.uab.edu) which includes RNA-sequencing data from 1,097 breast cancer and 114 normal tissues from the TCGA database. Additionally, the Kaplan-Meier plotter database (Nagy et al., 2021) (http://www.kmplot.com) was applied to estimate the affiliation between hub gene expression and prognosis in 1,090 breast cancer samples. Log-rank *p* < 0.05 was considered statistically significant. Lastly, a prognostic sub-network of ceRNA was constructed.

### Exploring the biological functions of prognosis-related genes

Using CancerSEA (Yuan et al., 2019) (http://biocc.hrbmu.edu.cn/CancerSEA/) database to explore the biological functions of prognosis-related genes in breast cancer. In addition, we analyzed the correlations between prognosis-related genes and proliferation markers (Ki-67, PCNA and MCM2) by Pearson correlation analysis. R represents correlation coefficient, which ranges between −1 and 1. The large R is, the greater the correlation between prognosis-related genes and proliferation markers. |R| > 0.4 and *p* < 0.05 were set as the cut-off criteria.

### Identification of potential gene-drug interaction

CTD database (Davis et al., 2021) (http://ctdbase.org) is an innovative database that provides information about gene-drug interactions, gene-pathways and gene-disease relationships. We developed a gene-drug network using the CTD database and Cytoscape 3.8.2 software to investigate associations between prognostic genes and chemotherapeutic drugs.

### Cell culture and transfection

The breast cancer cell line (MCF-7) and normal mammary epithelial cell line (MCF-10A) were purchased from the Cell Bank of Type Culture Collection of Chinese Academy of Sciences. MCF-7 cells were cultured in DMEM (Boster, Wuhan, China) supplemented with 10% fetal bovine serum (FBS) and 1% penicillin/streptomycin (Gibco, CA, United States). MCF-10A cells were kept in DMEM/F12 (Procell, Wuhan, China) supplemented with 20 ng/ml epidermal growth factor, 0.5 μg/ml hydrocortisone, 5% horse serum and 1% penicillin/streptomycin. Both cell lines were maintained in a humidified incubator at 37°C with 5% CO_2_.

Small interfering RNA (siRNA) targeting circ_0008812 (si-circ8812: ACG​AGU​GCA​CUU​GGU​GAA​AUU), circ_0001583 (si-circ1583: CAA​AGA​AGG​CCA​AGG​UUA​AUU) and negative control (si-NC) were generated by Tsingke Biotechnology (Chengdu, China). MCF-7 cells were transfected using Lipofectamine 3000 (Invitrogen) for 48 h.

### RNA extraction and quantitative real time polymerase chain reaction

Total RNA was extracted from cells using E.Z.N.A. Total RNA Kit I (Omega, United States), and reversely transcribed into cDNA by the RvertAid First Strand cDNA Synthesis Kit (Thermo Scientific, United States). Next, quantitative real time polymerase chain reaction (qRT-PCR) was performed using cDNA, Fast SYBR Green qPCR Master Mix (Exongen) and primers. All primers were synthesized by Tsingke Biotechnology (Chengdu, China) and listed in Table 2.

**TABLE 2:** | Primer sequences of RNAs used for qRT-PCR.

Gene ID	Forward primer (5′–3′)	Reverse primer (5′–3′)
GADPH	GGA​GCG​AGA​TCC​CTC​CAA​AAT	GGC​TGT​TGT​CAT​ACT​TCT​CAT​GG
hsa_circ_0008812	GCT​ACT​AGC​CCA​ACA​GCA​ACT	CCT​GCT​ACT​GGA​AAG​GCA​TCT
hsa_circ_0001583	AGC​GCT​AAG​AAA​ACA​CCG​AA	CCC​CAA​CTG​GCT​TCT​TAG​GTT

### MTT assay

Transfected MCF-7 cells were seeded into 96-well plates (5,000 cells/well). After 0, 24, 48, 72, 96 h of culture, 20 μl MTT solution (5 mg/ml) was added into each well, and incubated at 37°C for 4 h. Then, the formazan product was dissolved using 150 μl DMSO. Finally, the absorbance at 490 nm was measured using a microplate reader (Bio-Rad, CA, United States).

### Colony formation assay

MCF-7 cells were plated into 6-well plates (200 cells/well) and cultured for 14 day to form colony. After 14 day, 0.1% crystal violet solution (Biosharp, Beijing, China) was used to fix and stain colonies for 30 min. Next, the number of colonies was calculated.

### Statistical analysis

GraphPad Prism 8.0 was used for statistical analysis and graphing. All experiments were repeated at least 3 times, and data were presented as the mean ± standard deviation (SD) and compared using Student’s *t*-test. *p* < 0.05 was considered statistically significant.

## Results

### Identification of differentially expressed RNAs

**FIGURE 2:**
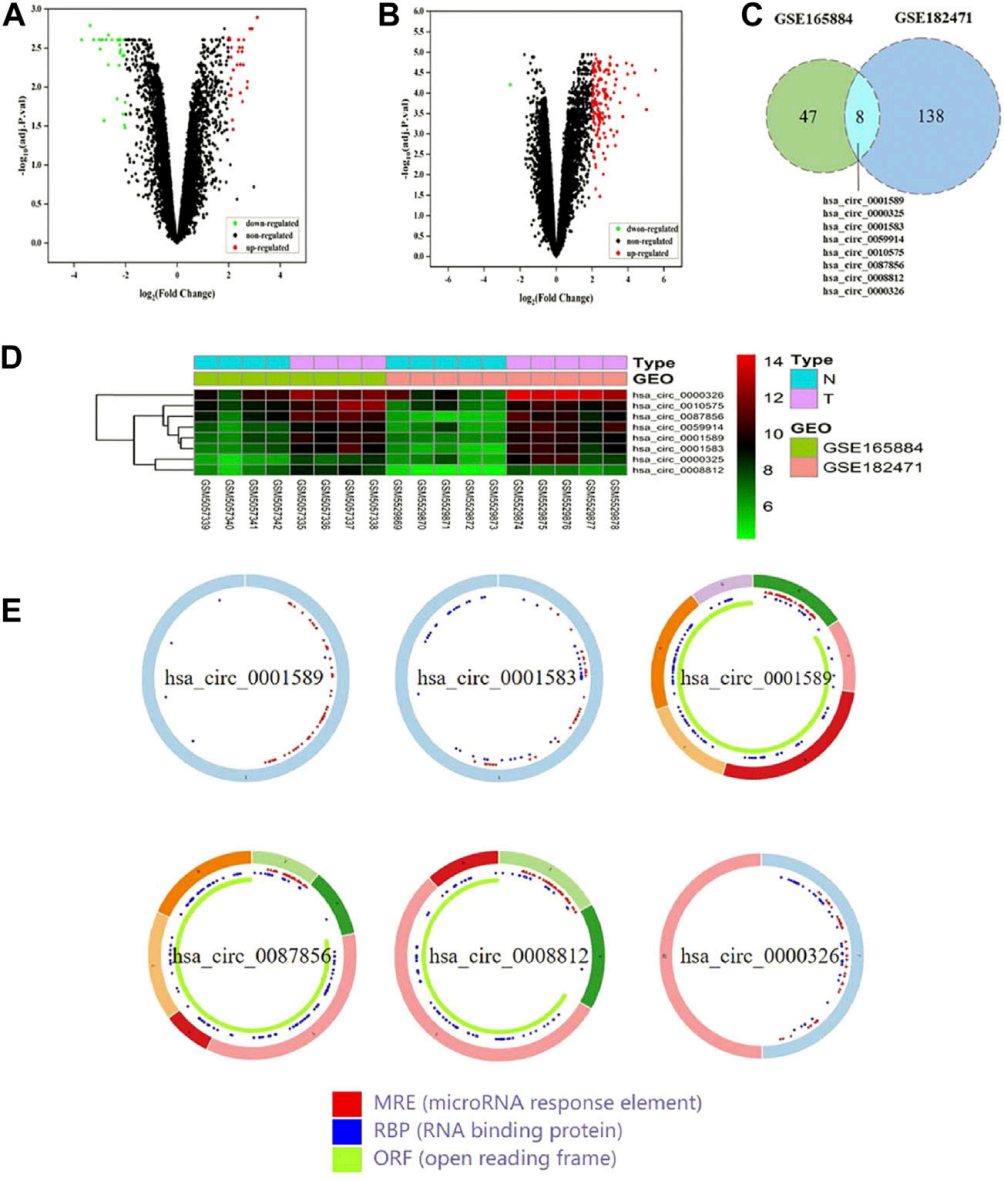
The identification of DEcircRNAs in two datasets. **(A)** Volcano plot of GSE165884. **(B)** Volcano plot of GSE182471. **(C)** Identification of overlapping DEcircRNAs from GSE165884 and GSE182471. **(D)** Heatmap of the overlapping 8 DEcircRNAs. **(E)** The structural patterns of the 6 DEcircRNAs from CSCD.

A total of 55 DEcircRNAs were identified from the GSE165884 dataset, including 28 up-regulated circRNAs and 27 downregulated ones (Figure 2A). A total of 146 DEcircRNAs were screened from the GSE182471 dataset, of which 145 circRNAs were upregulated and 1 circRNA was downregulated (Figure 2B). Eight overlapping DEcircRNAs were obtained from two datasets (Figures 2C,D), and Table 3 presents the specific information of these 8 DEcircRNAs, which enables us to better perform the following analyses, such as location on chromosomes, genome length and their expression in breast cancer. Based on data downloaded from TCGA database, we found 289 DEmiRNAs and 4914 DEmRNAs.

**TABLE 3:** Basic information of the 8 DEcircRNAs.

circRNA ID	Position	Genomic length	Strand	Best transcript	Gene symbol	Regulation
hsa_circ_0001589	chr6:26234499-26234709	210	−	NM_005320	HIST1H1D	Up
hsa_circ_0001583	chr6:26056122-26056299	177	−	NM_005319	HIST1H1C	Up
hsa_circ_0059914	chr20:32878137-32881962	3825	−	NM_001161766	AHCY	Up
hsa_circ_0010575	chr1:22157474-22207995	50521	−	NM_005529	HSPG2	Up
hsa_circ_0087856	chr9:110062421-110084399	21978	+	NM_002874	RAD23B	Up
hsa_circ_0008812	chr9:110062421-110074018	11597	+	NM_002874	RAD23B	Up
hsa_circ_0000326	chr11:65272490-65272586	96	+	TCONS_l2_00004572	TCONS_l2_00004572	Up
hsa_circ_0000325	chr11:65266845-65267149	304	+	TCONS_l2_00004575	TCONS_l2_00004575	Up

### Construction of circrna-miRNA-mRNA network

**FIGURE 3:**
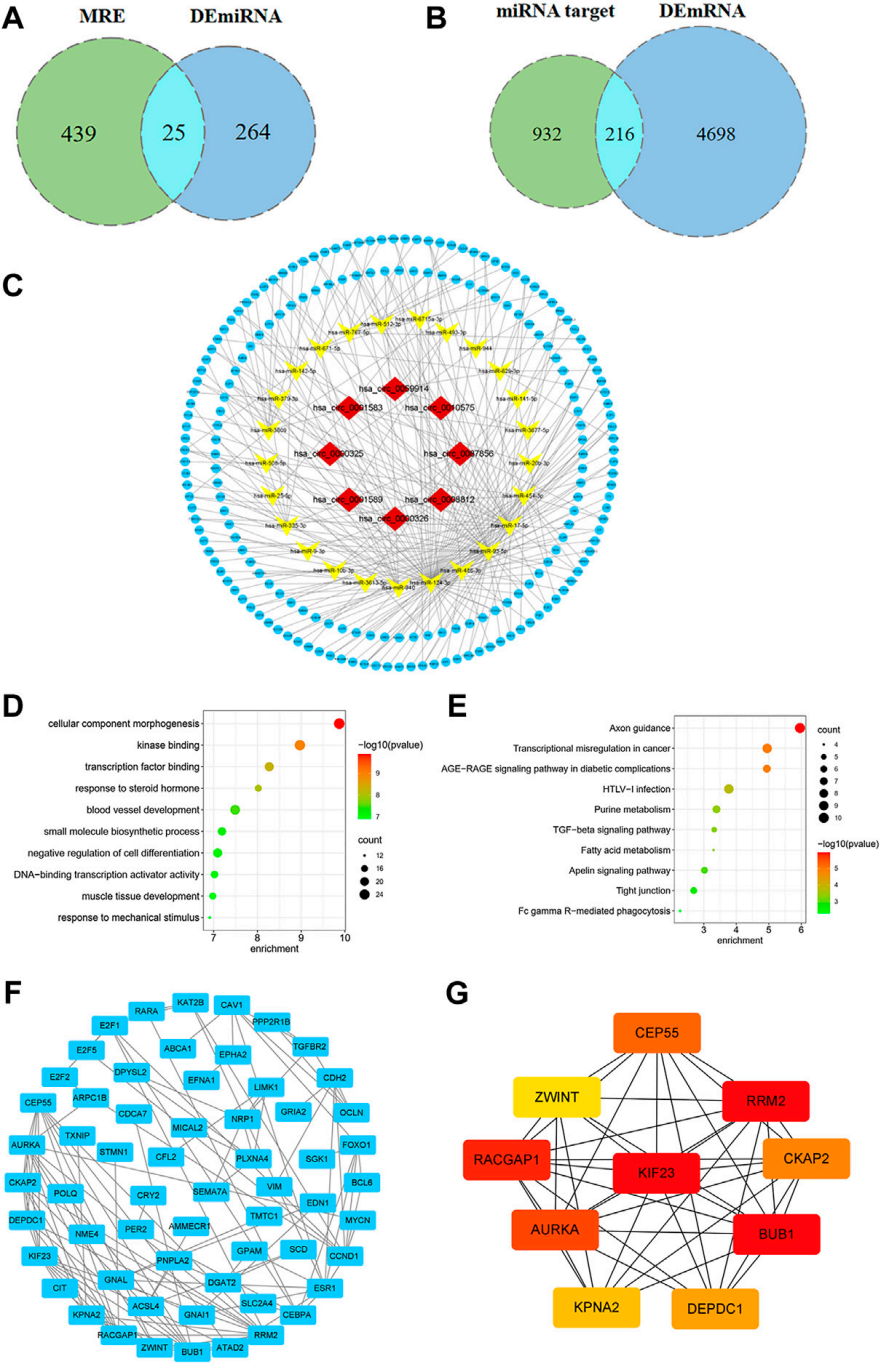
Construction of ceRNA network and identification of hub genes. **(A)** Venn diagram of overlapping miRNAs between circRNA-targeted miRNAs and DEmiRNAs. **(B)** Venn diagram of overlapping mRNAs between miRNA-targeted mRNAs and DEmRNAs. **(C)** The circRNA-miRNA-mRNA regulatory network in breast cancer. **(D)** GO analysis of candidate mRNAs. **(E)** KEGG pathway analysis of candidate mRNAs. **(F)** PPI network of 216 genes, consisting of 60 nodes and 117 edges. **(G)** PPI network of the top 10 hub genes.

Using the CSCD database, we predicted 303 circRNA-miRNA pairs. 2 out of the 8 DEcircRNAs, hsa_circ_0000325 and hsa_circ_0010575, which are not included in the CSCD database, were predicted in the CircInteractome database. Besides, the structural patterns of the 6 DEcircRNAs from CSCD database were shown in Figure 2E. A total of 161 circRNA-miRNA pairs were clarified. After intersecting with 289 DEmiRNAs, 32 circRNA-miRNA pairs were retained, including 8 circRNAs and 25 miRNAs (Figure 3A). Furthermore, we obtained 1,148 mRNAs targeted by these 25 miRNAs base on the miRDB, TargetScan and miRTarBase databases. After comparison with 4914 DEmRNAs, 216 overlapping mRNAs were selected as candidate mRNAs (Figure 3B). Finally, with Cytoscape software, we established the circRNA-miRNA-mRNA network. As shown in Figure 3C, the network consisted of 8 circRNAs, 25 miRNAs and 216 mRNAs.

### Gene ontology and kyoto encyclopedia of genes and genomes pathway analysis

Based on GO analysis, we found that these candidate mRNAs were associated with cellular component morphogenesis, kinase binding, transcription factor binding, small molecule biosynthetic process and DNA-binding transcription activator activity, etc. (Figure 3D). Furthermore, according to KEGG pathway analysis, mRNAs were found to be significantly enriched in axon guidance, transcriptional misregulation in cancer, purine metabolism, transforming growth factor-beta (TGF-β) and apelin signaling pathway (Figure 3E).

### Protein-protein interaction network analysis

After setting a composite score > 0.7 and removing unconnected nodes, we constructed a PPI network consisting of 60 nodes and 117 edges (Figure 3F). Moreover, we screened the top 10 hub genes from PPI network, which were KIF23, BUB1, RRM2, RACGAP1, AURKA, CEP55, CKAP2, DEPDC1, KPNA2 and ZWINT (Figure 3G).

### Validation of hub gene expression

**FIGURE 4:**
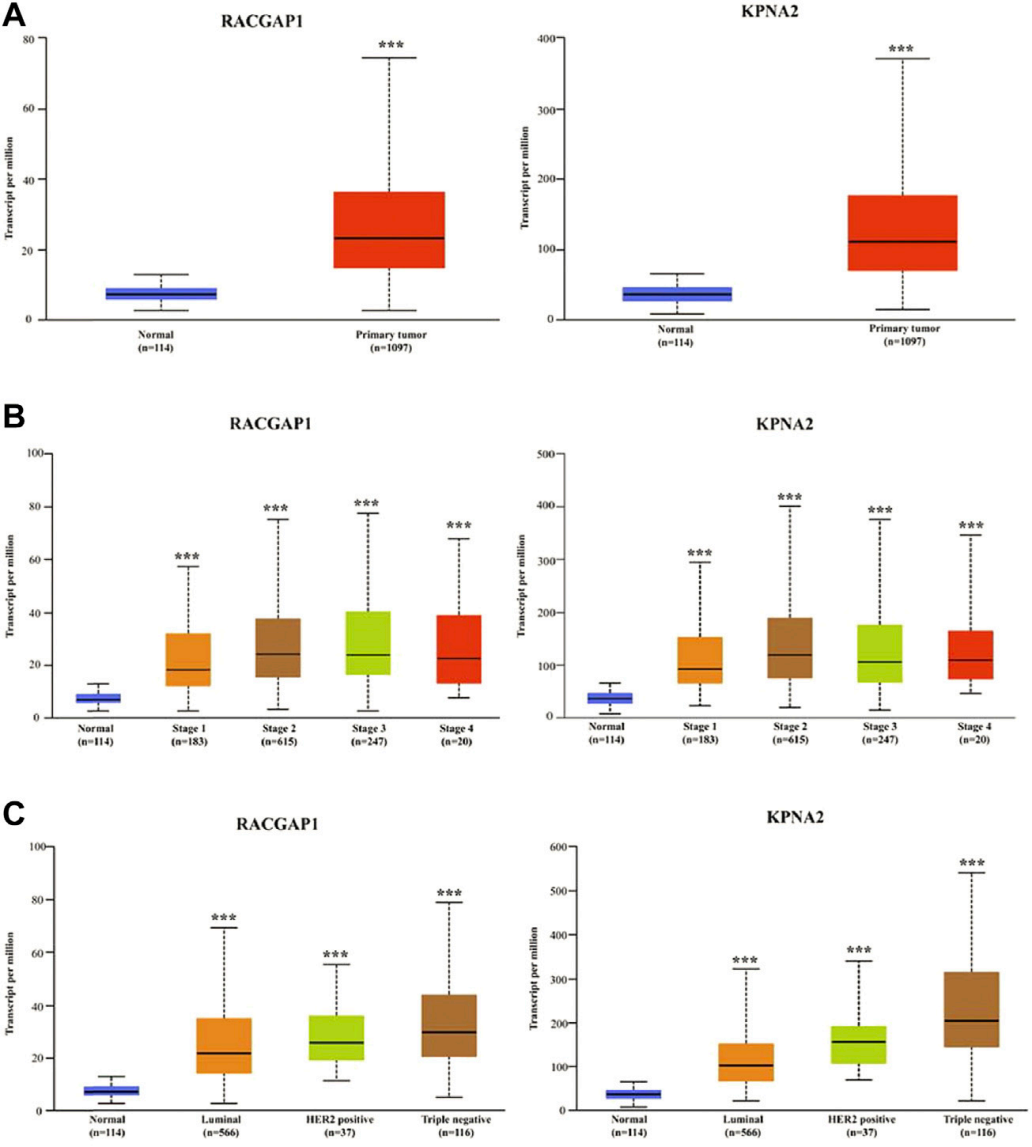
The validation of mRNA expression levels of hub genes. **(A)** The expression of hub genes in normal tissues and breast cancer tissues. **(B)** The mRNA levels of hub genes in normal tissues and breast cancer tissues at different stages. **(C)** The mRNA levels of hub genes in normal tissues and breast cancer tissues of different subclasses. ***p < 0.001.

Based on the UALCAN online database, the levels of these 10 hub genes were significantly elevated in breast cancer tissues compared with normal tissues (Figure 4A; Supplementary Figure S1). Furthermore, the differential expression of 10 hub genes between normal tissues and breast cancer tissues of different stages and subclasses was also investigated. As shown in Figure 4B; Supplementary Figure S2, the levels of these hub genes were significantly higher in both early and late tumor stages than that in normal tissues. Moreover, the expression of hub genes was significantly elevated in all three subtypes of breast cancer compared with normal tissues. The highest levels of RRM2 and ZWINT were found in HER2-positive breast cancer, and the highest expression of other hub genes was found in triple-negative breast cancer (Figure 4C; Supplementary Figure S3).

### Prognostic value of hub genes analysis

**FIGURE 5:**
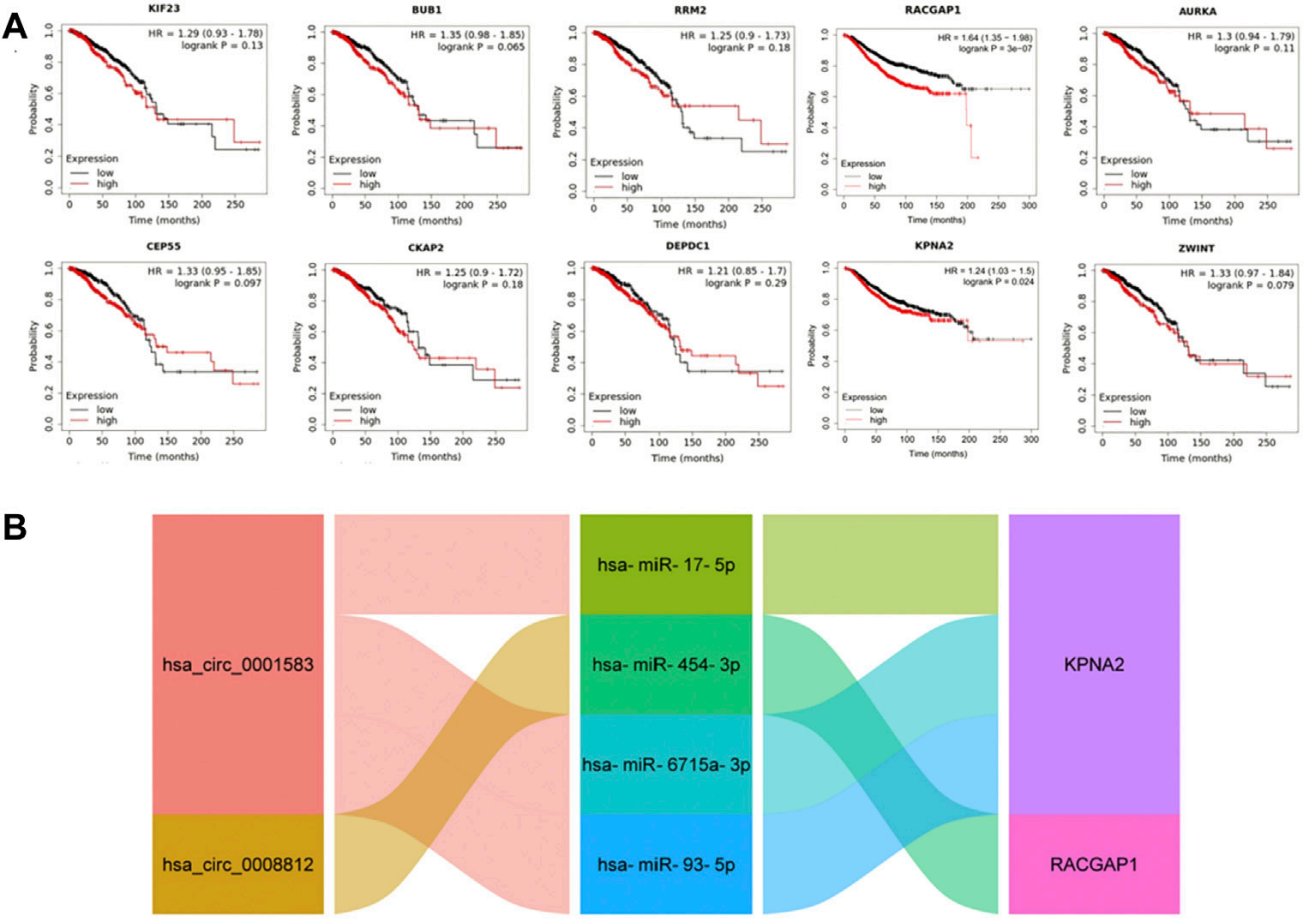
The construction of prognosis-related ceRNA network. **(A)** Kaplan-Meier survival analysis of hub genes. **(B)** The prognostic sub-network of ceRNA.

Using Kaplan-Meier plotter database, we found higher RACGAP1 [HR = 1.64 (1.35–1.98), logrank *p* = 3e-07] and KPNA2 [HR = 1.24 (1.03–1.5), logrank *p* = 0.024] mRNA levels were correlated with poorer prognosis (Figure 5A). We then established a prognostic sub-network of ceRNA based on the above results, including 2 circRNAs, 4 miRNAs and 2 mRNAs (Figure 5B).

### Biological functions of RACGAP1 and KPNA2

**FIGURE 6:**
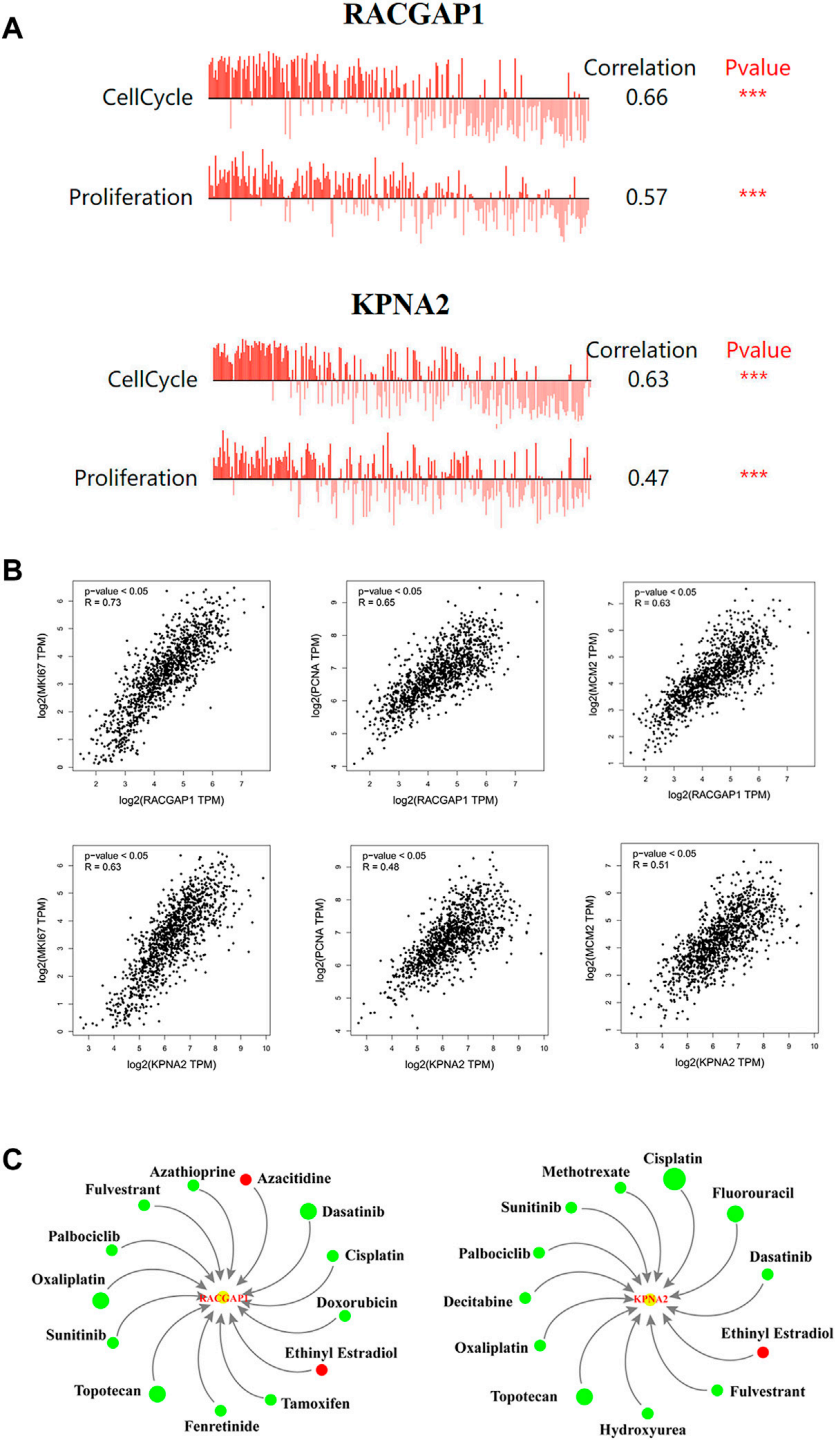
Biological functions of prognosis-related genes. **(A)** the biological functions of two prognosis-related genes in breast cancer. **(B)** the correlation prognosis-related genes and proliferation markers. **(C)** Gene-drug interaction network. Green circles: the drugs could reduce the expression of the two genes. Red circles: the drugs could elevate the expression of the two genes. The size of circles in the network represents the supported numbers of references. ***p < 0.001.

We found that the expression levels of RACGAP1 and KPNA2 in breast cancer cells were positively relevant to cell cycle and proliferation through CancerSEA database (Figure 6A). Similarly, their expression was positively correlated with Ki-67, PCNA and MCM2 expression (Figure 6B). The above results implied that RACGAP1 and KPNA2 had vital impacts on breast cancer progression.

### Gene-drug interaction network analysis

As shown in Figure 6C, numerous drugs could regulate these two prognosis-related genes, RACGAP1 and KPNA2. For instance, cisplatin, fulvestrant, palbociclib and oxaliplatin decreased RACGAP1 and KPNA2 levels, while ethinyl estradiol elevated their expression. Since high RACGAP1 and KPNA2 levels are associated with poor prognosis, this drug-gene interaction network might provide a reference for clinical rational drug use.

### Circ_0008812 and circ_0001583 promoted MCF-7 cell proliferation

**FIGURE 7:**
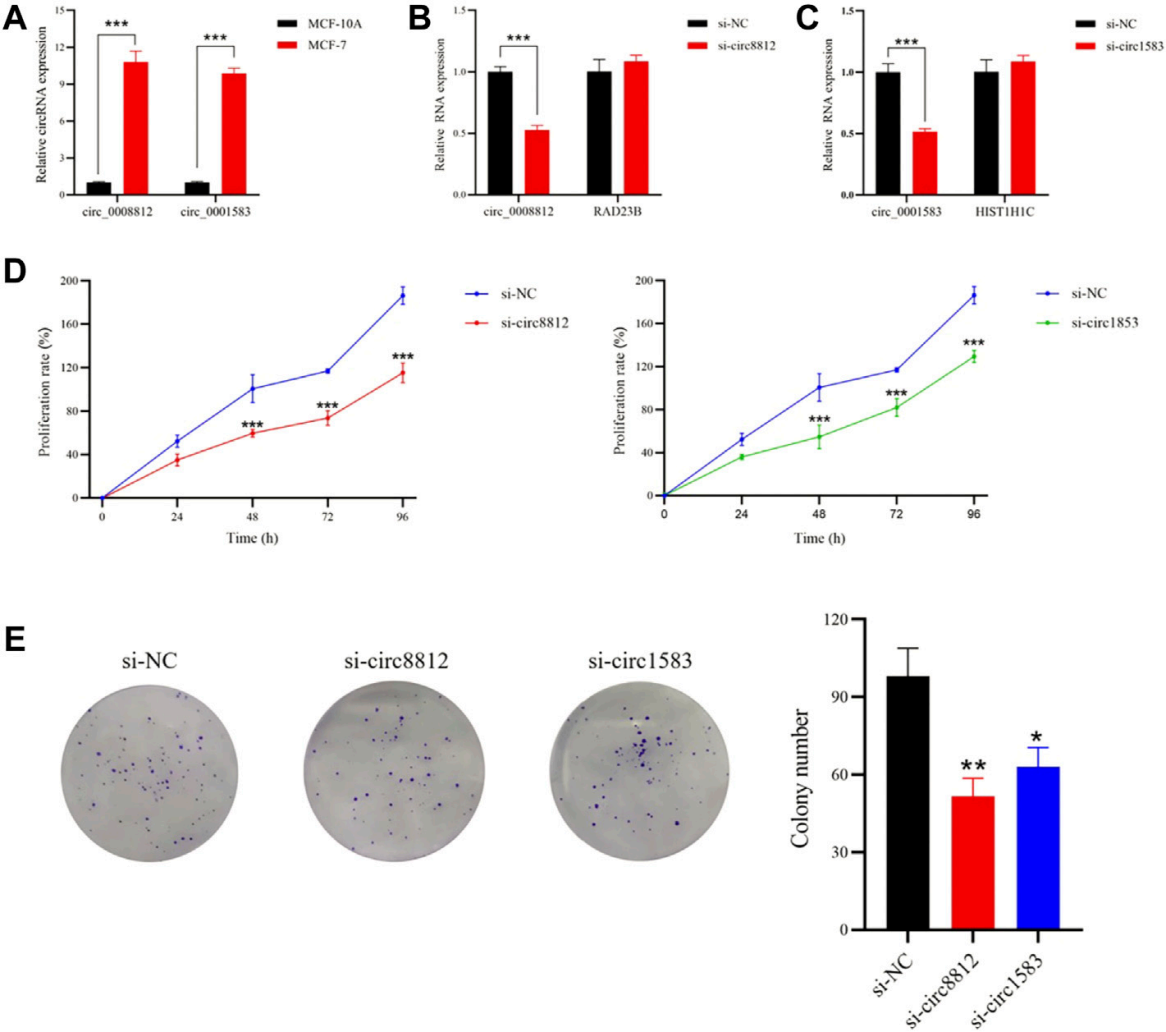
Circ_0008812 and circ_0001583 promoted MCF-7 Cell proliferation. **(A)** the expression of circ_0008812 and circ_0001583 was measured by qRT-PCR in MCF-10A and MCF-7 cell lines. **(B,C)** the expression levels of circ_0008812, circ_0001583 and thier parental genes in MCF-7 cells transfected with circ_0008812 and circ_0001583 siRNA. **(D,E)** the cell proliferation ability of MCF-7 cells treated with si-NC, si-circ8812 or si-circ1583 was detected by MTT and colony formation assay. The data are shown as mean ± SD of three independent experiments. *p < 0.05, **p < 0.01, ***p < 0.001.

Circ_0008812 and circ_0001583 were found to be highly expressed in MCF-7 cells by qRT-PCR, consistent with the GEO dataset results (Figure 7A). To detect the roles of circ_0008812 and circ_0001583 in MCF-7 cells, siRNAs were designed to knock down circ_0008812 and circ_0001583. qRT-PCR results showed that the expression of circ_0008812 and circ_0001583 was inhibited by siRNAs, without influencing their parental gene RAD23B and HIST1H1C expression (Figures 7B,C). MTT and colony formation assay revealed that depletion of circ_0008812 and circ_0001583 could significantly suppress MCF-7 cell proliferation (Figures 7D,E). These results implied that circ_0008812 and circ_0001583 might have essential impacts on the development of breast cancer.

## Discussion

In 1976, Sanger et al. first discovered circRNAs in viruses (Sanger et al., 1976), while circRNAs were previously considered to be by-products of pre-mRNAs (Cocquerelle et al., 1993; Wang and Fang, 2018).Compared with linear mRNAs, circRNAs are more stable in various species owing to their closed loop structures, which contribute to their resistance to RNases (Cao et al., 2021). Numerous circRNAs have been successfully characterized in diverse body fluids, such as plasma and saliva (Bahn et al., 2015; Ma et al., 2020). These findings highlighted that circRNAs could be considered as ideal biomarkers for various diseases. In recent decades, with the development of next-generation sequencing technology and bioinformatics, it has been confirmed that thousands of circRNAs exist in various species and participate in many biological processes. Additionally, accumulating evidence confirms that circRNAs play critical roles in the initiation and metastasis of malignant tumors (Zhang et al., 2019a; Xu et al., 2019). However, the regulatory mechanisms of circRNAs in tumors are still being explored. In this study, based on the GEO and TCGA database and a series of analyses, we found 2 functional circRNAs in breast cancer, which might be favorable to reveal the mechanisms of breast cancer initiation and development.

An increasing number of researches have shown that circRNAs possess biological functions through several mechanisms, including miRNA sponging, regulating parental gene transcription, interacting with proteins and being translated into proteins (Tang and Lv, 2021). For a long time, the function of translated proteins has been neglected due to the lack of the 5’ cap structure. Until 2017, Legnini et al. found that circ-ZNF609 could be translated into proteins in a cap-independent manner in mice, which provided evidence that circRNA could encode novel proteins/peptides in eukaryotes (Legnini et al., 2017). Acting as miRNA sponges is a key biological function of circRNAs involved in the regulation of malignant tumors (Hansen et al., 2013). Wu et al. (2021) clarified that circ_0000511 accelerated breast cancer progression by using miR-326 to elevate TAZ level. Dou’s group revealed the upregulation of circ_0008039 in breast cancer tissues and cells. Based on their research, it could positively mediate SKA2 expression via binding to miR‐140‐3p, leading to the development of breast cancer (Dou et al., 2021). However, many circRNAs in breast cancer still remain unidentified.

Several previous studies explored DEcircRNAs and ceRNA network in breast cancer based on online data and bioinformatics analysis (Zhao et al., 2019a; Sheng et al., 2021; Wang et al., 2021). For example, Sheng et al. constructed a circRNA-associated ceRNA network by performing circRNA microarray profile (GSE101123) from GEO database, miRNA and mRNA expression profiles from TCGA database (Sheng et al., 2021). However, they identified DEcircRNAs using only one circRNA dataset and did not reveal the functional roles of circRNAs in breast cancer.

In our current study, two latest circRNA microarray profiles (GSE165884 and GSE182471), miRNA and mRNA sequencing data were collected from GEO and TCGA databases. After differential expression analysis, 8 DEcircRNAs, 289 DEmiRNAs and 4914 DEmRNAs were identified. Among these 8 candidate circRNAs, hsa_circ_0000326 was reported to promote lung adenocarcinoma progression by modulating the miR-338-3p/RAB14 axis (Xu et al., 2020). Additionally, the expression of hsa_circ_0010575 in colon cancer tissues was significantly higher than that in normal ones (He et al., 2020). However, the possible role of hsa_circ_0010575 needs to be further explored. The other 6 DEcircRNAs (hsa_circ_0001589, hsa_circ_0001583, hsa_circ_0059914, hsa_circ_0087856, hsa_circ_0008812 and hsa_circ_0000325) have not been reported until now.

MiRNAs are a class of small non-coding RNAs that can participate in posttranscriptional regulation by targeting the 3’ untranslated region to inhibit target gene expression (Saliminejad et al., 2019). Over the past decades, many miRNAs have been shown to be dysregulated in cancers tissues and play vital roles in cancer onset, proliferation and metastasis (Ali Syeda et al., 2020). In our study, CSCD and CircInteractome databases were used to predict the sponge miRNAs of 8 DEcircRNAs. After intersecting with 289 DEmiRNAs, 25 miRNAs were obtained. Among them, 7 miRNAs (miR-671-5p, miR-940, miR-124-3p, miR-944, miR-93-5p, miR-512-3p and miR-142-5p) have been reported in breast cancer (Flores-Pérez et al., 2016; Tan et al., 2016; Wang et al., 2016; Zhang et al., 2020a; Elango et al., 2020; Mohamadzade et al., 2021; Pan et al., 2021). For instance, overexpression of miR-671-5p suppressed FOXM1 expression, thereby reducing breast cancer cells growth and invasion (Tan et al., 2016). MiR-124-3p could inhibit the proliferation of MCF-7 and MDA-MB-231 cells by suppressing CBL expression (Wang et al., 2016). Pan et al. (2021) showed that an increase in miR-93-5p could radiosensitize breast cancer cells by increasing apoptosis. Furthermore, we obtained 1,148 mRNAs targeted by these 25 miRNAs based on three online databases. 216 overlapping mRNAs were selected as candidate mRNAs after intersecting with 4914 DEmRNAs. Lastly, we set up a circRNA-miRNA-mRNA network.

Subsequently, functional enrichment analysis suggested that the mRNAs were primarily involved in kinase binding, transcription factor binding, transcriptional misregulation in cancer, regulation of TGF-β and apelin signaling pathway. Peng et al. (2015) found that long non-coding RNA (lncRNA) CASC2 might be involved in breast cancer metastasis by suppressing TGF-β pathway (Zhang et al., 2019b). It has been reported that miR-190 suppressed TGF-β-induced epithelial-mesenchymal transition via targeting SMAD2, thus inhibiting the metastasis of breast cancer cells, providing a new perspective to study the underlying mechanism of TGF-β pathway in tumors (Yu et al., 2018). Peng’s group showed that upregulation of apelin-13 in breast cancer tissues could expedite MCF-7 cells proliferation and invasion by regulating ERK_1/2_/AIB1 pathway (Peng et al., 2015).

To investigate the key genes in mammary carcinogenesis, we established a PPI network and screened out 10 hub genes, including KIF23, BUB1, RRM2, RACGAP1, AURKA, CEP55, CKAP2, DEPDC1, KPNA2 and ZWINT. These 10 hub genes have been reported to have significant effects on cancers (Zhang and Zhao, 2017; Huang et al., 2018; Zhao et al., 2019b; Ge et al., 2019; Luo et al., 2019; Wang-Bishop et al., 2019; Li et al., 2020b; Yang et al., 2020; Zhu et al., 2020; Jian et al., 2021). Moreover, their expression was higher in breast cancer tissues than in normal tissuses, in both early and late tumor stages, as well as in the three subtypes of breast cancer. Kaplan-Meier survival analysis suggested that elevated RACGAP1 and KPNA2 levels were significantly associated with poor prognosis. Additionally, their expression was positively correlated with that of Ki-67, PCNA and MCM2, which was consistent with the predictions of CancerSEA, implying that RACGAP1 and KPNA2 could facilitate breast cancer progression. Gene-drug interaction analysis showed that many chemotherapeutic drugs, such as doxorubicin, cisplatin, tamoxifen, fulvestrant and oxaliplatin, could decrease the mRNA expression levels of RACGAP1 and KPNA2. The results implied that RACGAP1 and KPNA2 might be a promising target in breast cancer therapy in the future. RACGAP1 is a member of the Rho GTPase-activating proteins family, and is essential in cytokinesis induction (Saigusa et al., 2015). Moreover, RACGAP1 has been found to affect cell apoptosis, proliferation, transformation and inflammation (Imaoka et al., 2015; Wang et al., 2018). In a clinical study, RACGAP1 mRNA expression was measured in high-risk breast cancer patients. Univariate analysis illustrated that elevated RACGAP1 level was inversely associated with overall survival (OS) and disease-free survival (DFS) (Pliarchopoulou et al., 2013). Moreover, the authors performed Cox multivariate regression analysis and found that high level of RACGAP1 may act as a disadvantageous prognostic factor for death and independently predict poor OS. Ren et al. (2021) found that RACGAP1 has a positive effect on breast cancer metastasis by regulating epithelial cell transforming 2-dependent mitochondrial quality control. KPNA2 belongs to the nuclear transporter family (Han and Wang, 2020). Many studies have shown that KPNA2 might serve as a potential predictor for cancer prognosis (Huang et al., 2013; Li et al., 2013; Zeng et al., 2021). Zhang et al. (2020b) revealed that a lncRNA LINC00461 could positively modulate KPNA2 by sponging miR-144-3p, thus promoting breast cancer cells invasion and migration (Duan et al., 2020). A recent study revealed that depletion of KPNA2 suppressed breast cancer exacerbation via blocking NF-κB signaling and nuclear translocation of c-Myc (Zhang et al., 2020b).

CeRNA prognostic sub-networks composed of 2 circRNAs, 4 miRNAs and 2 mRNAs were constructed based on RACGAP1 and KPNA2. By qRT-PCR, circ_0008812 and circ_0001583 belonging to this prognostic sub-network were uncovered to be upregulated in MCF-7 cells. Knockdown of circ_0008812 and circ_0001583 decreased the proliferation ability of MCF-7 cells. These results implied that circ_0008812 and circ_0001583 might modulate breast cancer initiation and development through 4 axes, including miR-4554-3p/RACGAP1, miR-93-5p/KPNA2, miR-6715a-3p/KPNA2 and miR-17-5p/KPNA2. Nevertheless, this assumption needs further verification.

However, there are some limitations in our study. Firstly, GSE165884 and GSE182471 contain 4 and 5 paired cancer and normal tissues, respectively. The sample sizes are small and the datasets containing more samples are needed to validate our findings. In addition, our preliminary results uncovered the functions of circ_0008812 and circ_0001583 in breast cancer, and the in-depth mechanisms will be elucidated in our future studies.

In conclusion, we constructed 4 prognostic regulatory axes based on a comprehensive analysis of the GEO and TCGA databases, including circ_0008812/miR-4554-3p/RACGAP1, circ_0001583/miR-93-5p/KPNA2, circ_0001583/miR-6715a-3p/KPNA2 and circ_0001583/miR-17-5p/KPNA2 regulatory axes. Furthermore, depletion of circ_0008812 and circ_0001583 inhibited the proliferation of breast cancer cells, indicating that these circRNAs have oncogenic roles. Our study provides an advanced perspective on the mechanisms of breast cancer initiation and development, which might help to find promising breast cancer prognostic biomarkers.

## Data Availability

The original contributions presented in the study are included in the article/Supplementary Material, further inquiries can be directed to the corresponding author.

## Supplementary material

SUPPLEMENTARY FIGURE S1 The mRNA levels of hub genes in breast cancer and adjacent normal tissues.

SUPPLEMENTARY FIGURE S2 The mRNA levels of hub genes in normal tissues and breast cancer tissues at different stages.

SUPPLEMENTARY FIGURE S3 The mRNA levels of hub genes in normal tissues and breast cancer tissues of different subclasses.
