# Supplementary figures and images for: Upregulation of circ_0008812 and circ_0001583 predicts poor prognosis and promotes breast cancer proliferation
Source: Front Mol Biosci. 2022 Sep 19;9:1017036. doi: 10.3389/fmolb.2022.1017036 (PMC9527282; doi:10.3389/fmolb.2022.1017036)

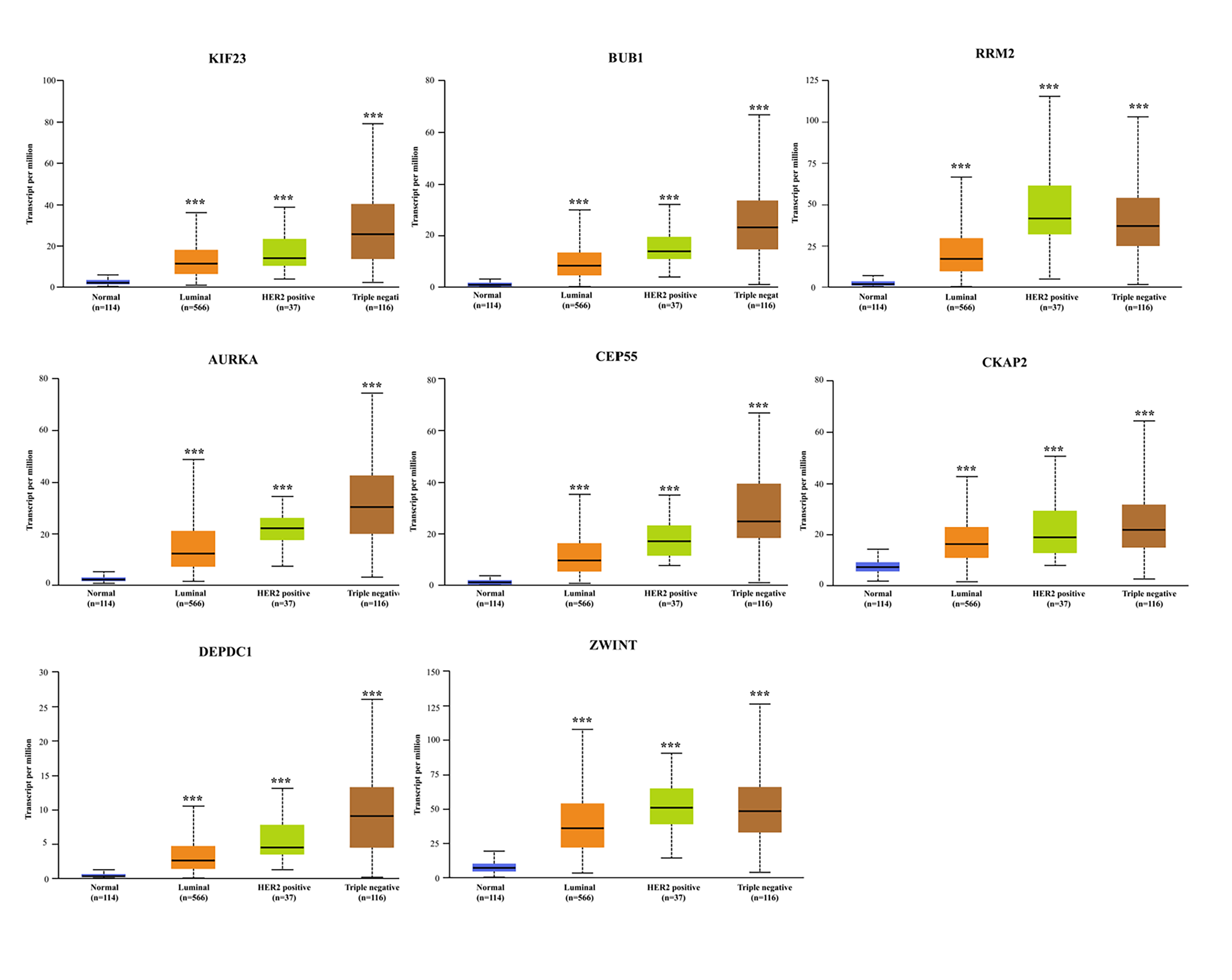

Supplement: Supplementary file 1 [file Image3.TIF]

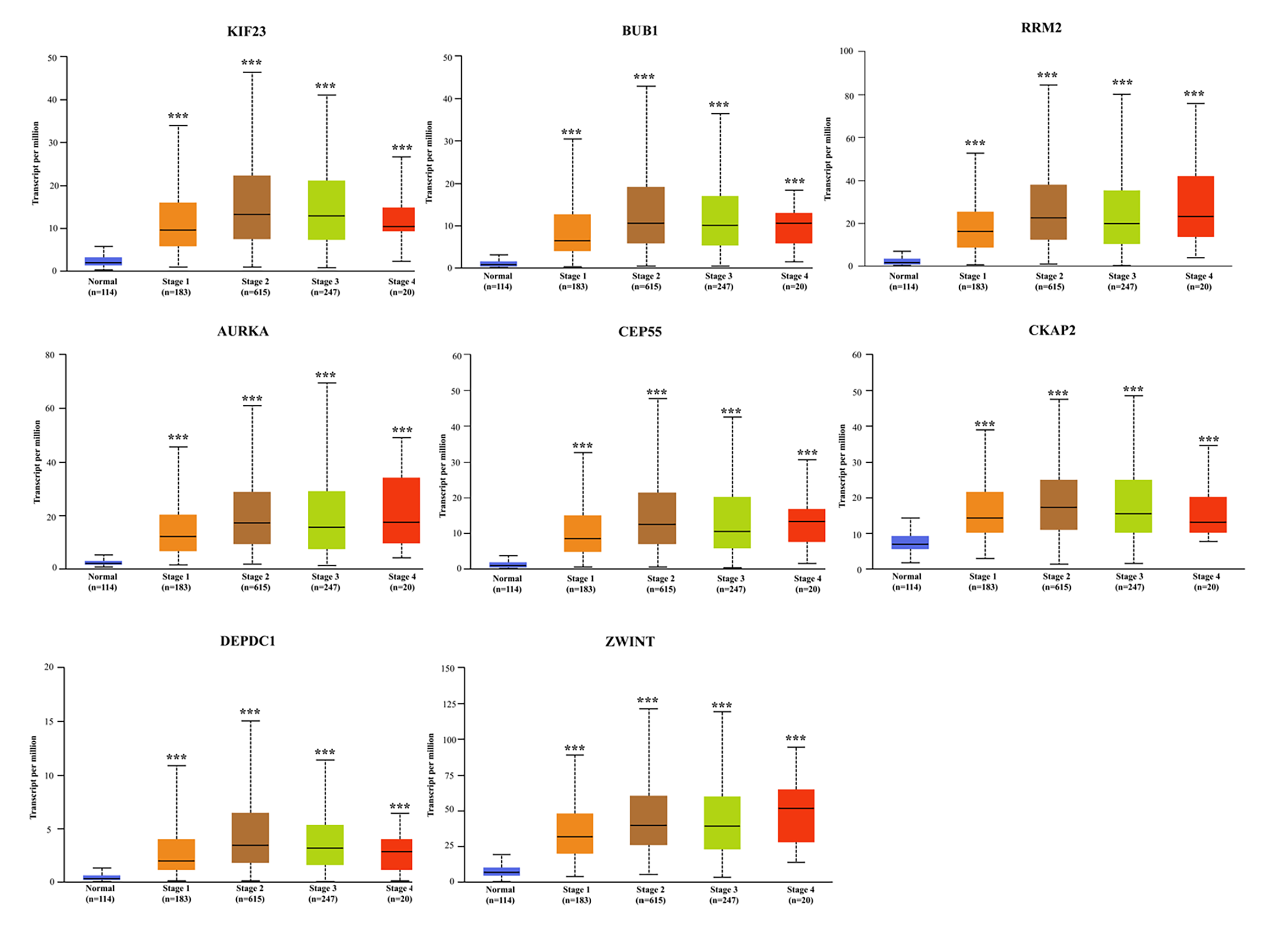

Supplement: Supplementary file 2 [file Image2.TIF]

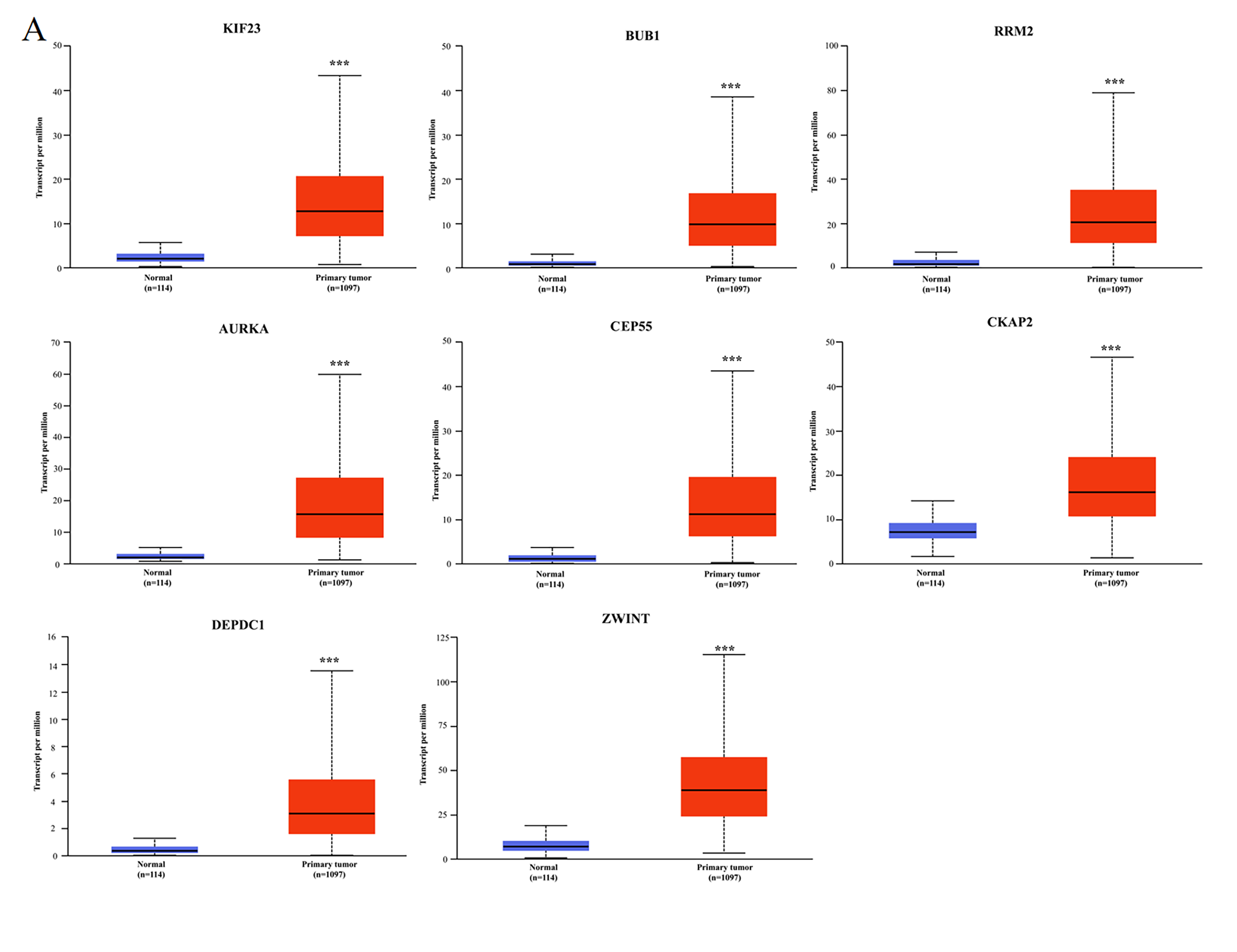

Supplement: Supplementary file 3 [file Image1.TIF]
